# Determination of Radiation Absorbed Dose to Primary Liver Tumors and Normal Liver Tissue Using Post-Radioembolization ^90^Y PET

**DOI:** 10.3389/fonc.2014.00255

**Published:** 2014-10-13

**Authors:** Shyam M. Srinivas, Navin Natarajan, Joshua Kuroiwa, Sean Gallagher, Elie Nasr, Shetal N. Shah, Frank P. DiFilippo, Nancy Obuchowski, Bana Bazerbashi, Naichang Yu, Gordon McLennan

**Affiliations:** ^1^Department of Nuclear Medicine, Cleveland Clinic, Cleveland, OH, USA; ^2^Case Western Reserve University School of Medicine, Cleveland, OH, USA; ^3^Ohio University Heritage College of Osteopathic Medicine, Athens, OH, USA; ^4^Department of Quantitative Health Sciences, Cleveland Clinic, Cleveland, OH, USA; ^5^Section of Vascular and Interventional Radiology, Cleveland Clinic, Cleveland, OH, USA; ^6^Department of Radiation Oncology, Cleveland Clinic, Cleveland, OH, USA

**Keywords:** Yttrium-90, radioembolization, HCC, ^90^ Y PET/CT, dosimetry, response

## Abstract

**Background:** Radioembolization with Yttrium-90 (^90^ Y) microspheres is becoming a more widely used transcatheter treatment for unresectable hepatocellular carcinoma (HCC). Using post-treatment ^90^ Y positron emission tomography/computerized tomography (PET/CT) scans, the distribution of microspheres within the liver can be determined and quantitatively assessed. We studied the radiation dose of ^90^ Y delivered to liver and treated tumors.

**Methods:** This retrospective study of 56 patients with HCC, including analysis of 98 liver tumors, measured and correlated the dose of radiation delivered to liver tumors and normal liver tissue using glass microspheres (TheraSpheres^®^) to the frequency of complications with modified response evaluation criteria in solid tumors (mRECIST). ^90^ Y PET/CT and triphasic liver CT scans were used to contour treated tumor and normal liver regions and determine their respective activity concentrations. An absorbed dose factor was used to convert the measured activity concentration (Bq/mL) to an absorbed dose (Gy).

**Results:** The 98 studied tumors received a mean dose of 169 Gy (mode 90–120 Gy; range 0–570 Gy). Tumor response by mRECIST criteria was performed for 48 tumors that had follow-up scans. There were 21 responders (mean dose 215 Gy) and 27 non-responders (mean dose 167 Gy). The association between mean tumor absorbed dose and response suggests a trend but did not reach statistical significance (*p* = 0.099). Normal liver tissue received a mean dose of 67 Gy (mode 60–70 Gy; range 10–120 Gy). There was a statistically significant association between absorbed dose to normal liver and the presence of two or more severe complications (*p* = 0.036).

**Conclusion:** Our cohort of patients showed a possible dose–response trend for the tumors. Collateral dose to normal liver is non-trivial and can have clinical implications. These methods help us understand whether patient adverse events, treatment success, or treatment failure can be attributed to the dose that the tumor or normal liver received.

## Introduction

### Hepatocellular carcinoma

Hepatocellular carcinoma (HCC) affects approximately 500,000 people worldwide, representing the third leading cause of cancer related deaths and the sixth most prevalent cancer (1, 2). Axelrod and von Leeuwen have reported that the incidence of HCC has “more than doubled, from 2.6 to 5.2 per 100,000 population” over the past 20 years, with an increase in mortality from 2.8 to 4.7 per 100,000 (1). Owing to this increase in incidence is the earlier acquisition of hepatitis B and C in conjunction with high-risk behavior (1). Additionally, the obesity epidemic has contributed to an increase in non-alcoholic steatohepatitis (NASH), which can eventually progress to fibrosis, cirrhosis, and HCC (1).

### Current treatments

The treatment of HCC has been problematic, since most patients present at an advanced stage (3). In early stages of the disease, surgical treatments – primarily resection and transplantation – provide the best curative outcomes. Ideal candidates for resection are those patients with small solitary tumors and preserved liver function, whereas patients who meet the Milan criteria and who are not good candidates for resection often benefit from liver transplantation (2). A disadvantage of resection, however, is that patients’ remnant livers may not be able to support the necessary hepatic functional demands, and there is a high potential for recurrent disease (4).

Other treatment modalities include transarterial chemoembolization (TACE), sorafenib, external beam radiation, and radiofrequency ablation. In comparison to sorafenib and external beam radiation, more local therapies such as radiofrequency ablation, radioembolization, and TACE are able to deliver the desired dose to the target with minimal toxicity to the system (5). Supporting this statement, Dezarn et al. noted that the maximum external beam acceptable dose to the whole liver of 35 Gy delivered in 1.8 Gy/day fractions is far below the 70 Gy typically needed to destroy solid tumor lesions (6). The sensitivity of normal hepatic tissue to external beam radiation has given way to more locally effective techniques. Stereotactic body radiotherapy (SBRT), therefore, is usually reserved for those patients who have well-defined tumors on MRI or CT, and the ability to treat safely is limited by lesion number, distribution, and location (6).

An emerging and innovative practice in the treatment of HCC is the transcatheter angiographic delivery of Yttrium-90 (^90^Y) microspheres that is indicated for metastatic colorectal carcinoma (CRC) as well as HCC (5). Typically, under fluoroscopic guidance, the arterial catheter is optimally placed and the radiolabeled particles injected into the vascular distribution of the tumor. Injection through the hepatic artery provides ^90^Y treatment with an advantage over other modalities, since hepatic malignancies receive approximately 70–80% of their blood supply via the hepatic artery (6). The injected particles then become trapped at the precapillary level, and emit internal [β] radiation, providing more localized, higher dose delivery as compared to external beam radiation (5). At the same time, surrounding normal liver tissue that is perfused primarily from portal vein circulation is relatively preserved.

The Radioembolization and Brachytherapy Oncology Consortium (REBOC) recommends that because the nature of primary and secondary hepatic malignancies differs, therapy should be tailored to the disease (7). For example, according to one study, multinodular HCC without vascular invasion needs to be treated by transarterial chemoembolization, while HCC with vascular invasion or distant metastasis is suited for treatment with sorafenib, a targeted multikinase inhibitor administered orally (8). Additionally, according to REBOC, patients with liver metastases from other primary sites should be offered systemic therapy before ^90^Y treatment, and in the case of primary hepatic tumors, patients should undergo hepatology and transplant evaluations to determine the optimal treatment strategy (7). A study by Gulec et al. demonstrated that ^90^Y microsphere selective internal radiation treatment effectively controls tumor growth, with a possible induction of contralateral lobe hypertrophy (4).

### Yttrium-90

Intravascular delivery of the radioactive isotope yttrium-90 has been practiced since the mid 1960s (9). The use of microspheres embedded with a β-emitting radionuclide, such as Yttrium-90, has been available to investigators in the United States since 2000 (6, 7).

^90^Y is produced by neutron bombardment of Y-89 in a commercial reactor. ^90^Y is a beta radiation emitter having average energy of 0.94 MeV, with a mean tissue penetration of 2.5 mm, and a maximum range of 1.1 cm. One gigabecquerel of ^90^Y delivers approximately 49.38 Gy/kg to tissue. The half-life of ^90^Y is 2.67 days or 64.2 h (7).

### ^90^Y positron emission tomography/computerized tomography

While many studies have supported the benefits and advantages of HCC treatment with ^90^Y, there are multiple factors that must be considered. In a review of the relevant literature, Pasciak et al. have noted that ^90^Y positron emission tomography/computerized tomography (PET/CT) is capable of providing accurate information with regard to the microsphere distribution in a lesion. They also noted that activity concentration for ^90^Y can be quantified with or without time-of-flight acquisition. However, despite known methods to calculate dose, there is no standardized method of dosimetric measurement (10).

In early clinical trials, it was assumed that the activity and distribution of the ^90^Y particles were uniform throughout the liver. However, recent analyses of livers that had been imaged or explanted have demonstrated that uniform distribution is not the case (11). Due to the radiolucency of the ^90^Y microspheres, real-time monitoring of treatment and distribution is not possible during the infusion process (12). To address this, methods to monitor distribution of ^90^Y have been developed.

Kao et al. note that the distribution of microsphere therapy within the target arterial territory is dependent upon locoregional flow environment distal to the point of injection, the injection rate, the timing interval, proximity to branching daughter vessels, extent of lung shunting, cardiovascular status, and particle load (13). Their study compared the previously held gold-standard for post-^90^Y microsphere radioembolization imaging, ^90^Y Bremsstrahlung SPECT/CT, with ^90^Y PET/CT. The study’s conclusion was that ^90^Y PET/CT is superior to ^90^Y Bremsstrahlung SPECT/CT for the assessment of target and non-target activity (13).

Zade et al. demonstrated the superiority of ^90^Y PET/CT over ^90^Y Bremsstrahlung SPECT/CT. While β radiation emitted by ^90^Y interacts with body tissues resulting in Bremsstrahlung radiation, which has conventionally been used for imaging the biodistribution of this isotope, the wide energy range of Bremsstrahlung radiation makes imaging involving ^90^Y technically challenging (12). Elschot et al. have also demonstrated that ^90^Y PET/CT is superior to Bremsstrahlung SPECT/CT because of its superior resolution and lack of specialized techniques for quantification of images while utilizing comparable image acquisition time (14).

Owing to the success of ^90^Y PET/CT, other researchers have used this valuable tool as a means of developing a dose calculation technique based on the observed distribution of microspheres after infusion (15, 16). Others have reported a 40-Gy increase in absorbed dose to tumor and complete resolution of disease in the treated area within 3 months by optimizing treatment from observed ^90^Y PET/CT activity (17, 18).

The purpose of this study is to utilize ^90^Y PET/CT to determine the dosimetry to tumors and normal liver post-radioembolization treatment and the relationship of dose to outcome.

## Materials and Methods

### Phantom experiment

The IEC body phantom experiment was performed in order to assess the PET camera’s ability to image ^90^Y quantitatively. Scans were performed on day 0, day 3, day 5, and day 7 after loading the phantom with a liquid ^90^Y chloride solution using a PET/CT camera (Siemens Biograph mCT) having 22 cm PET axial field of view and capable of time-of-flight PET. CT images were obtained immediately prior to the PET scan for the purpose of attenuation correction and scatter correction. Prior to imaging, the phantom was prepared using ^90^Y chloride solution with activity (3.15 GBq) calibrated by the supplier (Perkin-Elmer, Waltham, MA, USA). An abdominal-shaped container was filled to capacity with 9.717 mL of sterile water in the background compartment mixed with 10 g DTPA. Six spheres (of diameters 37, 28, 22, 17, 13, and 10 mm) located on the container’s center plate were filled with ^90^Y solution to approximately an 8:1 ratio, and a 3D acquisition and reconstruction protocol of the phantom was created. The protocol included two bed positions with duration of 20 min per position. Since ^90^Y PET is a rather novel scan procedure, the scanner software did not allow the selection of ^90^Y, thus Ge-68 was selected instead, with correction for the relative positron branching fractions made later (see below). The PET reconstruction protocol utilized the 3D OSEM algorithm including time-of-flight information and point spread function modeling with 2 iterations, 21 subsets, 8 mm Gaussian filter, and zoom factor 1.0. The reconstruction included corrections for attenuation, scatter, random coincidences, detector normalization, and dead time.

The result of the phantom experiment revealed that our Biograph mCT PET scanner was able to estimate the known ^90^Y activity filled in the spheres to an average accuracy of −4.8% [range −15–2.2%] for the spheres of 37 and 28 mm diameter.

### Overview of patients treated and calculation of normal liver activity

In this Institutional Review Board approved and Health Insurance Portability and Accountability Act compliant retrospective study, 56 HCC patients treated with TheraSpheres^®^ (BTG, London, UK) were studied between December 7, 2010 and May 22, 2013. The main purpose was to evaluate the radiation absorbed dose by tumors and the radiation absorbed dose by the region of normal non-malignant liver tissue that is incidentally treated by ^90^Y microsphere therapy. Both glass and resin microspheres are used at our center but only glass microspheres (TheraSpheres^®^) were included in this study. The ^90^Y activity was prescribed according to the manufacturer recommended guidelines, using the standard MIRD-based formula [Dose = 49.38 (Activity/mass)].(7) Our institutional practice is to give the ^90^Y activity that will result in each lobe of the liver receiving ~120 Gy. Using a specialized imaging program from MIM software (Cleveland, OH, USA), contours were drawn on CT images to highlight and isolate tumor volumes, as well as the volumes of liver treated by ^90^Y microspheres. The software was used to identify the volume of the contoured region, as well as the activity concentration of the contoured region in Bq/mL using the contour statistics tool. The MIM software allowed us to determine volumes and activity concentration for the entire treated liver region and the tumor regions, but the subdivision described below, modified slightly from what has been presented in the literature (19), needed to be employed to evaluate the activity concentration of the normal non-malignant liver tissue that was also contained in the treated region.

The following subdivision is assumed, where *A*_x_ represents the total activity of the region *x* in Bq:

(1) $$A_{\text{Treated liver}}=A_{\text{Tumor}}+A_{\text{Normal liver}}$$

Since *A*_x_ = (*C*_x_)(*V*_x_), where *C*_x_ is the activity concentration of region *x* in Bq/mL and *V*_x_ is the volume of the region *x* in mL, Eq. 1 can be rewritten as:

(2) $$\begin{array}{llll} \left(V_{\text{Treated liver}}\right)\left(C_{\text{Treated liver}}\right) & =\left(V_{\text{Tumor}}\right)\left(C_{\text{Tumor}}\right)\; & & \;\\ & \;+\left(V_{\text{Normal liver}}\right)\left(C_{\text{Normal liver}}\right) \end{array}$$

Algebraic rearrangement of Eq. 2 with the use of the identity (*V*_Normal liver_) = (*V*_Treated liver_) − (*V*_Tumor_) provides us with the activity concentration of normal liver:

(3) $$\left(C_{\text{Normal liver}}\right)=\frac{\left(V_{\text{Treated liver}}\right)\left(C_{\text{Treated liver}}\right)-\left(V_{\text{Tumor}}\right)\left(C_{\text{Tumor}}\right)}{\left(V_{\text{Treated liver}}\right)-\left(V_{\text{Tumor}}\right)}$$

If multiple tumors were present, then (*V*_Tumor_)(*C*_Tumor_) in Eq. 3 was replaced by the following equation:

(4) $$\begin{array}{llll} \left(V_{\text{Tumor}}\right)\left(C_{\text{Tumor}}\right) & \;=\;\left(V_{\text{Tumor 1}}\right)\left(C_{\text{Tumor 1}}\right)+\left(V_{\text{Tumor 2}}\right)\left(C_{\text{Tumor 2}}\right)\; & & \;\\ & \;+\left(V_{\text{Tumor 3}}\right)\left(C_{\text{Tumor 3}}\right)... \end{array}$$

Following activity concentration determination, activity concentration (Bq/mL) for tumor and normal liver were converted to an absorbed dose (Gy) by using an absorbed dose factor that will be discussed later in the Section “^90^Y absorbed dose calculation”.

### Treated liver volume and activity determination

**Figure 1:**
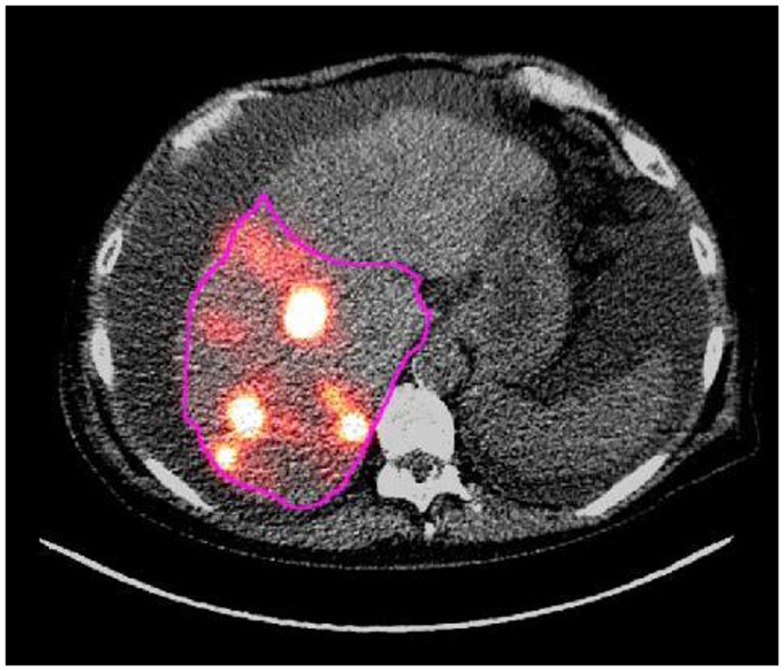
**This image shows a contour in pink drawn around a treated region in an axial slice of the AC CT/PET fusion image that was acquired following right lobe ^90^Y microsphere therapy**.

The attenuation correction CT (AC CT) that is acquired with the PET scan following ^90^Y radioembolization was used to contour the volume of treated liver (i.e., right lobe or left lobe). The ^90^Y PET scan and the accompanying AC CT were analyzed using MIM software. A fusion of the PET and AC CT was viewed in order to visualize which regions of the liver were targeted and contained the most activity. The treated lobe volume was contoured on the AC CT using a contour tool in MIM. Contours were individually drawn in the axial view of the AC CT. Contours were drawn on each plane to ensure that the entire treated region was encompassed. The region of treatment was determined by examining the ^90^Y PET activity distribution on the fusion image and also by viewing medical records of the ^90^Y microsphere therapy procedure that indicated the intended target liver lobes for therapy. After the contours were drawn, the contour statistics tool in MIM was used to display the volume of the contour region in mL and the activity concentration of the contour region in Bq/mL (see Figure 1). Three trainees contoured both liver and tumor volumes under the supervision of a board certified nuclear medicine physician.

### Tumor characterization and deformation

The triphasic abdominal CT scans that were acquired most recently prior to ^90^Y microsphere therapy were used to contour tumor volumes. The tumor volume was visualized in either the arterial or venous phase CT, and the tumor was contoured. Contours were individually drawn in the axial view of the either arterial phase obtained 30 s post-injection or the venous phase obtained 60 s post-injection. Multiple contours were used if multiple tumors were present. Tumors that were present in the liver but not in the intended region of therapy were not contoured. The tumor regions were determined by examination of the arterial and venous phase CT scans and were confirmed by reading the CT imaging reports with respect to specific information about hepatic morphology and tumor location.

**Figure 2:**
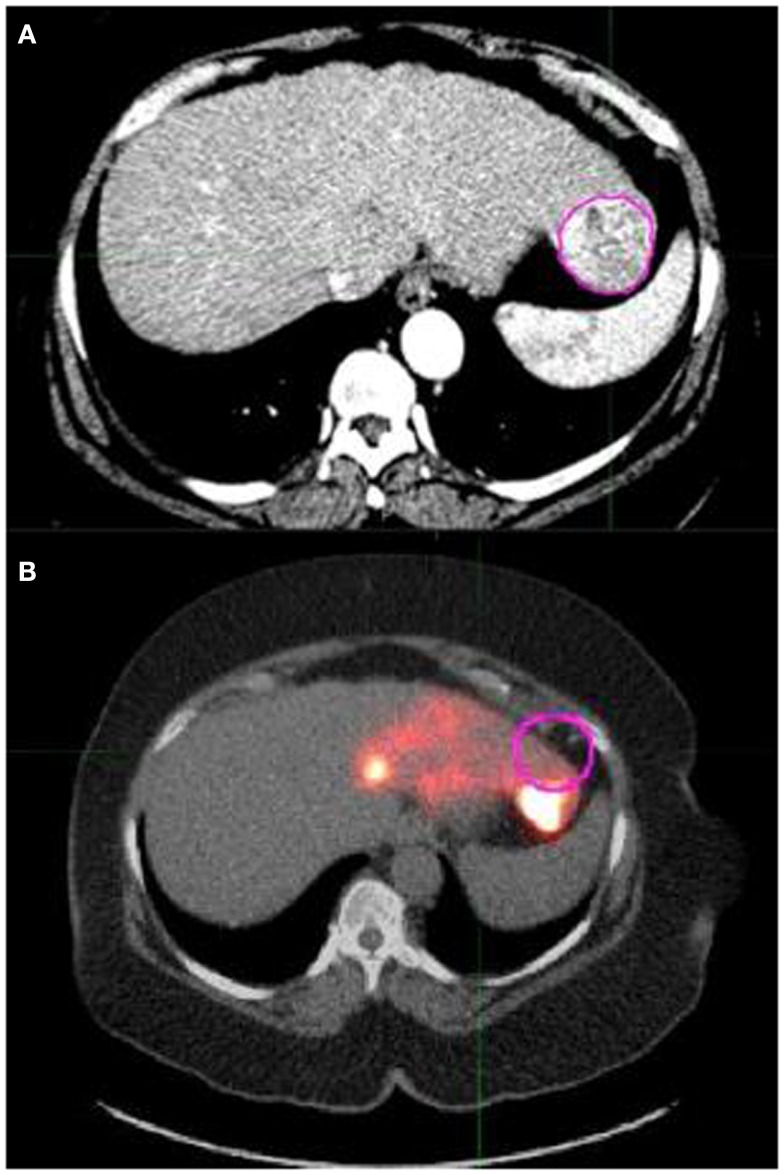
**(A)** shows a pink contour drawn around a tumor in an axial slice of the arterial phase abdominal CT scan. **(B)** shows the same tumor contour overlaid on the AC CT/PET fusion image that was acquired following left lobe ^90^Y microsphere therapy. **(B)** exhibits that contours drawn on the arterial CT show poor overlap with the corresponding region in the AC CT/PET fusion image. This can lead to inaccurate activity concentration determinations and necessitated the need to deform the arterial CT scan for more accurate contour statistic calculations.

The ^90^Y PET/CT scan, which was required for determination of tumor radioactivity, typically showed poor overlap with the arterial and venous phase CT scans due to variations in position of patient, free breathing vs. breath hold, and time gap between the two acquisitions (see Figure 2). For this reason, the abdominal CT scan on which the tumor contours were drawn was deformed to match the AC CT corresponding to the PET scan using the deformation tools in MIM. The “reg refine” tool in MIM allowed us to fix points of interest that should be conserved in the deformation process. These points were mostly fixed around the liver edges since this was our organ of interest. Around 60 points were fixed around the liver in multiple planes and then the local alignments were converted into a deformable registration using the MIM deformation tool. The tumor contours drawn on the abdominal CT were deformed in the process as well.

**Figure 3:**
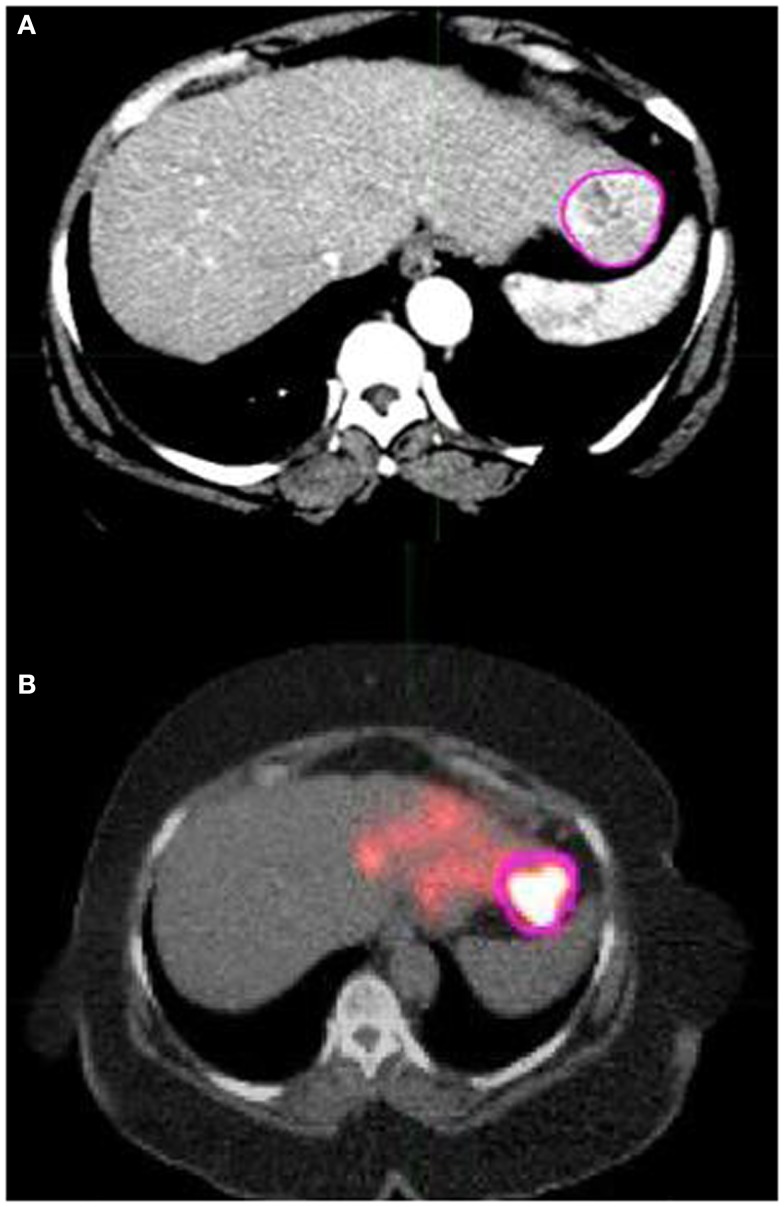
**(A)** shows a pink contour drawn around the tumor in an axial slice of the deformed version of the arterial phase CT scan that was shown in Figure 2A. **(B)** shows the same tumor contour overlaid on the same AC CT/PET fusion scan shown in Figure 2B that was acquired following left lobe ^90^Y microsphere therapy. **(B)** exhibits that contours drawn on the deformed arterial CT show good overlap with the corresponding region of interest in the AC CT/PET fusion image. Because this allows for more accurate contour statistic calculations, the deformation tools in MIM were utilized.

This process provided a deformed abdominal CT scan that showed increased similarity of liver morphology to the AC CT/PET fusion scan, which ensured more accurate determination of tumor volumes and radioactivities compared to the undeformed scan (see Figure 3). The compute “contour statistics” tool evaluated the contoured tumor region and displayed the volume of the contour region in mL and the mean activity concentration in Bq/mL. Multiple sets of values were obtained if multiple tumors were present. The mean activity concentration (Bq/mL) of the tumor can be converted to an absorbed dose (Gy) of the tumor by using the absorbed dose factors that were determined from branching ratios as explained in the next section.

### ^90^Y absorbed dose calculation

Our calculation utilized dosimetry based on a modification to the local deposition model using ^90^Y post-radioembolization dose (11, 20, 21) as opposed to technetium-99m macroaggregated albumin SPECT images (18, 22). Following determination of the mean activity concentration of the tumors and normal liver, the activity concentration in Bq/mL needed to be converted to absorbed doses of ^90^Y in units of Gy. On the PET acquisition workstation, the radionuclide Ge-68 was selected since ^90^Y was not available for selection. Ge-68 is long lived (half-life of 270.8 days) and decays to Ga-68, which is short lived (half-life of 68 min) and has high positron branching (0.891). The radioactivity concentration measured by the PET scanner (*C*_Ge_) is in terms of Ge-68/Ga-68 positron events and has units of Bq/mL. The first step was the conversion of the radioactivity concentration in terms of Ge-68/Ga-68 positron events into a radioactivity concentration in terms of ^90^Y positron events (*C*_Y_), which is also in units of Bq/mL. This was done using the ratio of branching coefficients for Ge-68/Ga-68 and ^90^Y (23).

(5) $$\frac{\left(C_{\text{Ge}}\right)\left(0.\text{891 }\beta^{+}∕\text{Ge-68 decay}\right)}{\left(0.0000\text{316 }\beta^{+}{∕}^{90}\text{Y decay}\right)}\;=C_{\text{Y}}$$

The *C*_Y_ was then divided by a factor of 10^6^ and multiplied by the absorbed dose factor 49.38 in order to convert Bq/mL into units of Gy (6). Decay was taken into account between the time of injection of ^90^Y and the time of PET scan. The overall conversion of the radioactivity concentration in terms of Ge-68/Ga-68 decay (*C*_Ge_) that is determined by the scanner, into a dose of absorbed ^90^Y (D^90^_Y_) can be represented by the following equation:

(6) $$\frac{\left(C_{\text{Ge}}\right)\left(0.\text{891 }\beta^{+}∕\text{Ge decay}\right)\left(\text{49}.\text{38}\right)e^{\text{t}\left(\text{ln2}\right)∕\text{64}.\text{1}}}{\left(0.0000\text{316 }\beta^{+}∕\text{Y decay}\right)\left(\text{1}0^{\text{6}}\right)}\;=D_{90_{\text{Y}}}$$

This formula, nearly identical to the conversion factor used by Pasciak et al. (21), was used for all radiation dose calculations for both tumors and normal liver.

### Statistical methods

Tumor response was divided into two categories for analysis: treatment responders vs. non-responders. Responders were defined as complete response, partial response, or enough remittance of tumor burden to enable a liver transplant. Treatment non-responders were defined as stable disease, progressive disease, or if the patient died before the treatment response could be assessed. A logistic regression model was built at the tumor level. The dependent variable was the modified response evaluation criteria in solid tumors (mRECIST) outcome dichotomized as responders vs. non-responders. The independent variable was the tumor (Gy) in the liver segment. Generalized estimating equations (GEEs) were used to account for the correlation in multiple lesions from the same patient. A significance level of 0.05 was used to test for an association between dose and the probability of response.

An additional analysis was performed to explore a possible correlation between dose of radiation to normal liver tissue and complications suffered within 2 months of ^90^Y radioembolization. Complications included abnormal liver function tests, right upper quadrant pain, ascites, cholecystitis, or physician notes indicating poor response and functional status of the patient following therapy. Those patients who had only slight fatigue and slight abdominal pain were grouped with patients who had no complications. The remaining complications were considered severe. Logistic regression models were built to predict severe complications as a function of Gy delivered to normal liver tissue. The dependent variable was the presence/absence of severe complications; the independent variable was the Gy to the normal liver. A significance level of 0.05 was applied to test the effect of dose.

## Results

**Figure 4:**
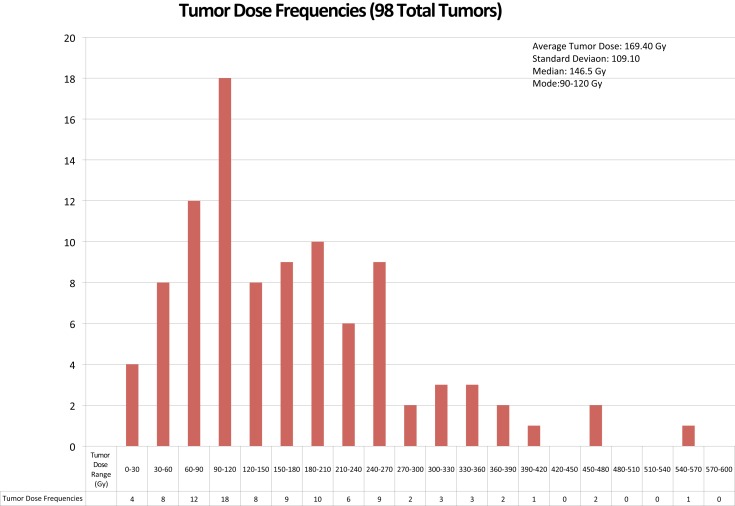
**The above chart displays the frequencies of radiation doses delivered to tumor tissue in the 56 cases of ^90^Y radioembolization therapy for hepatocellular cancer that were analyzed**.

**Figure 5:**
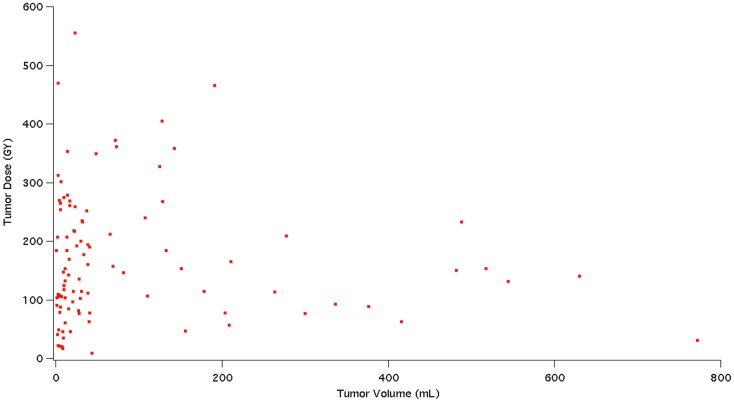
**This figure shows a scatter plot representation of tumor dose (Gy) in relation to tumor volume (mL)**.

The analysis on our cohort suggests that the mean dose received among the 98 studied tumors was 169 Gy with a mode of 90–120 Gy and a treatment dose range of 0–570 Gy. The overall distribution of doses delivered to tumor is displayed in Figure 4. Additionally, Figure 5 indicates the relationship between tumor dose and tumor volume. This scatter plot shows the inherent difficulty in achieving a substantial Gy in a tumor of large volume. If one were to consider >100 Gy to be a treatment goal (6), then clearly the majority of the tumors in which this was achieved were smaller than 100 mL in volume.

Modified response evaluation criteria in solid tumors were used to assess the overall tumor response to radioembolization therapy. mRECIST guidelines state that lesions are suitable for mRECIST criteria if they can be classified as a RECIST measurable lesion, the lesion is suitable for repeat measurement, or the lesion shows intratumoral enhancement on contrast-enhanced CT or MRI. Target lesion responses to therapy were classified as complete (CR), partial (PR), stable (SD), or progressive disease (PD) based on their respective change in size and contiguous intratumoral enhancement on arterial phase CT or MRI. Size change measurements were based on the longest enhancing tumor diameter per mRECIST guidelines. The mRECIST analysis was performed by a medical student with oversight from an abdominal radiologist. Lesions qualified as a CR if there was a disappearance of intratumoral arterial enhancement (vis-a-vis total necrosis). PR involved a 30% decrease in the diameters of viable target lesions, whereas progressive disease demonstrated an increase of at least 20% in the diameters of viable lesions. SD did not meet the criteria for either a PR or PD (24).

Fifty-seven of the 98 tumors that were assessed for dosage were deemed acceptable to be assessed for tumor response. They met the following characteristics: hyper-enhancing on arterial phase of CT or MRI, hypo-enhancing on venous phase of CT or MRI, and size greater than or equal to 2 cm. If <2 cm, they had to show organ procurement and transplantation network (OPTN) 5A criteria to ensure accurate analysis as well as reduce quantitative errors, which could arise from smaller lesion sizes as shown by Willowson et al. (25).

The transplant recipient’s tumors (*n* = 2, mean 142 Gy) and the deceased patients’ tumors (*n* = 7, mean 221 Gy) were unable to be assessed by mRECIST due to not having post-treatment images. Thus, data analysis was performed on the 48 tumors in 33 patients who were able to be stratified with an mRECIST score, revealing 21 responders and 27 non-responders with a mean tumor (Gy) of 215 and 167, respectively (see Tables 1 and 2). The association between mean tumor (Gy) and response was not statistically significant (*p* = 0.099) (Table 3) but was suggestive of a trend for greater likelihood of a response as tumor (Gy) increased.

**Table 1:** **The below table illustrates the responses of 23 separate tumors deemed to be “responders” after ^90^Y radioembolization treatment resulted in complete or partial response or enough remittance of tumor burden to enable a liver transplant**.

^90^Y Tumor responders (tumor response categorized by mRECIST)					
Liver segment(s)	Mean tumor (Gy)	Tumor size before treatment (cm)	Tumor size at follow-up (cm)	Elapsed time to follow-up (months)	Overall response
Left lobe	114	1.7	2.6 (all necrotic)	2.6	Complete
5,8	372	5.8	3.2	5.9	Partial
2	104	2.5	1.7	3.7	Partial
3	190	4.6	2.6	3.7	Partial
6,7	79	2.2	1.5	3.0	Partial
2	469	1.9	1.1 (all necrotic)	4.5	Complete
3	87	1.9	all necrotic	4.5	Complete
8	147	4.1	4.2 (all necrotic)	4.5	Complete
6	555	3.8	1.0	3.3	Partial
2	349	4.4	2.9 (all necrotic)	5.9	Complete
8	207	1.8	3.2 (all necrotic)	5.5	Complete
8	17	2.2	1.0	3	Partial
7	269	3.6	1.9	3	Partial
6	131	6.4	4.0	5.9	Partial
6	405	4.3	1.0	1.9	Partial
1	259	2.8	1.6	4.1	Partial
4	153	2.3	2.7 (all necrotic)	7.3	Complete
5	30	11.8	3.7	7.3	Partial
4B	115	4.6	0.9	2.7	Partial
2,3	158	6.5	4.1	2.6	Partial
2	301	2	0.9 (all necrotic)	2.5	Complete
8	22	1.2	–	–	Liver transplant
5	261	1.7	–	–	Liver transplant

*The overall responses were based on mRECIST criteria. Of these 23 tumors that were deemed to be responders, 8 had complete response, 13 had partial response, and 2 had remittance of disease that allowed liver transplant*.

**Table 2:** **The below table illustrates the responses of 34 separate tumors deemed to be “non-responders” after ^90^Y radioembolization**.

^90^Y Tumor non-responders (tumor response categorized by mRECIST)					
Liver segment(s)	Mean tumor (Gy)	Tumor size before treatment (cm)	Tumor size at follow-up (cm)	Elapsed time to follow-up (months)	Overall response
Lateral right lobe	209	3.2	3.1	7.5	Stable
4A,8	465	5.3	10.2	7.3	Progressive disease
8	78	5.1	5.3	3	Stable
2	212	2.2	1.6	1.6	Stable
Right lobe	46	1.5	1.5	4.3	Stable
5	362	4.2	3.4	8.1	Stable
6	279	13.1	13.4	8	Stable
8	200	3.9	3.7	4.4	Stable
Right lobe	233	9.4	8.3	7.6	Stable
2,3	207	3.2	4.2	2.3	Progressive disease
2	124	3.7	3.1	2.3	Stable
4B	270	1.8	2	2.3	Stable
8	275	2.2	2.4	2.3	Stable
8	35	2	2.2	3	Stable
6	233	3.2	2.8	3	Stable
Right lobe	63	9.7	8.6	5.8	Stable
6	103	3	2.7	5.3	Stable
6	78	3.6	3.4	6.9	Stable
7	234	3.1	3.1	2.6	Stable
7	46	2.6	2.5	2.7	Stable
8	76	2.8	2.8	2.6	Stable
Left lobe	92	9.9	7.5	3.1	Stable
5	117	4.2	3.7	4.4	Stable
7	62	5.3	4.8	4.8	Stable
8	217	2.1	2.5	4.8	Stable
6	160	2.6	3.2	1.4	Progressive disease
6	40	4.1	3.9	7.5	Stable
Right lobe	153	8.6	–	–	Patient deceased
6, 7	327	5.6	–	–	Patient deceased
8	146	5	–	–	Patient deceased
Left lobe	169	1.3	–	–	Patient deceased
8	353	1.9	–	–	Patient deceased
7	265	2.2	–	–	Patient deceased
6	132	3	–	–	Patient deceased

*For this study, non-responders were defined by stable disease, progressive disease, or if the patient died before the treatment response could be assessed. The overall responses were based on mRECIST criteria. Of these 34 tumors that were deemed to be non-responders, 24 had stable disease, 3 had progressive disease, and 7 tumors were unable to be assessed for response due to death*.

**Table 3:** **Statistical analysis of the 48 tumors evaluable by mRECIST criteria, which had follow-up scans available**.

Outcome (*n*)	Mean tumor dose (Gy)	Standard deviation (Gy)	Dose range (Gy)
CR (*n* = 8)	228	133	87–469
PR (*n* = 13)	206	160	17–555
SD (*n* = 24)	154	96	35–362
PD (*n* = 3)	277	164	160–465
Responders (CR and PR)	215		
Non-responders (SD and PD)	167	*p* = 0.099	

*Patients who went to transplant or died were excluded. There were 21 responders and 27 non-responders. The association between mean tumor (Gy) and the response may indicate a trend, but was not statistically significant (*p* = 0.099)*.

Tumor response was divided into two categories for analysis: treatment responders vs. non-responders. Responders were defined as complete response, partial response, or enough remittance of tumor burden to enable a liver transplant. Twenty-three tumors were found to be responders per this definition (see Table 1). Treatment non-repsonders were defined as stable disease, progressive disease, or if the patient died before the treatment response could be assessed. Thirty-four tumors were found to be treatment non-responders per this definition (see Table 2). Data analysis done on the 48 tumors in 33 patients who were able to be stratified with an mRECIST score revealed 21 responders and 27 non-responders with a mean tumor absorbed dose of 215 and 167 Gy, respectively. A logistic regression model was built at the liver segment level. The dependent variable was the mRECIST outcome dichotomized as CR and PR as responders, and SD and PD as non-responders. The independent variable was the tumor (Gy) in the liver segment. GEEs were used to account for the correlation in multiple segments from the same patient. A significance level of 0.05 was used to evaluate the effect of tumor dosage on outcome. The association between mean tumor absorbed dose and response did not reach statistical significance due to a *p*-value of 0.099 (see Table 3). The transplant recipient’s tumors (*n* = 2, mean 142 Gy) and the deceased patients’ tumors (*n* = 7, mean 221 Gy) were unable to be assessed by mRECIST due to not having post-treatment images.

**Figure 6:**
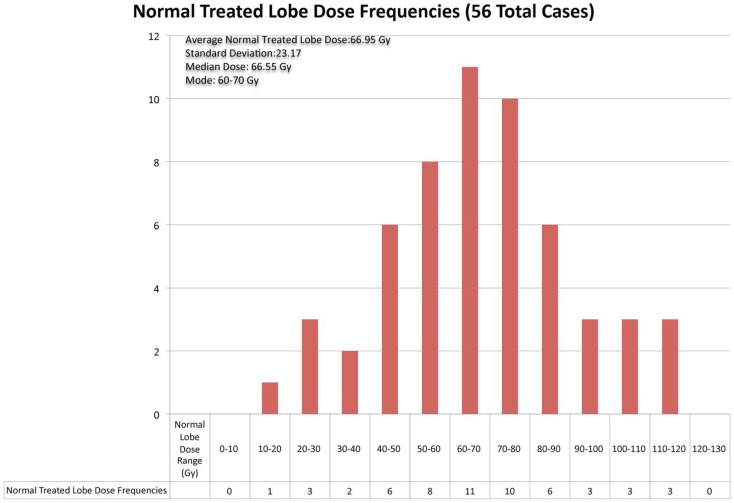
**The above chart displays the frequencies of radiation doses delivered to normal liver tissue in the 56 cases of ^90^Y radioembolization therapy for hepatocellular cancer that were analyzed**.

Normal liver tissue in the 56 cases evaluated received a mean absorbed dose of 67 Gy with a mode of 60–70 Gy and a range of 10–120 Gy. The overall distribution of doses delivered to normal treated liver lobe is displayed in Figure 6.

**Figure 7:**
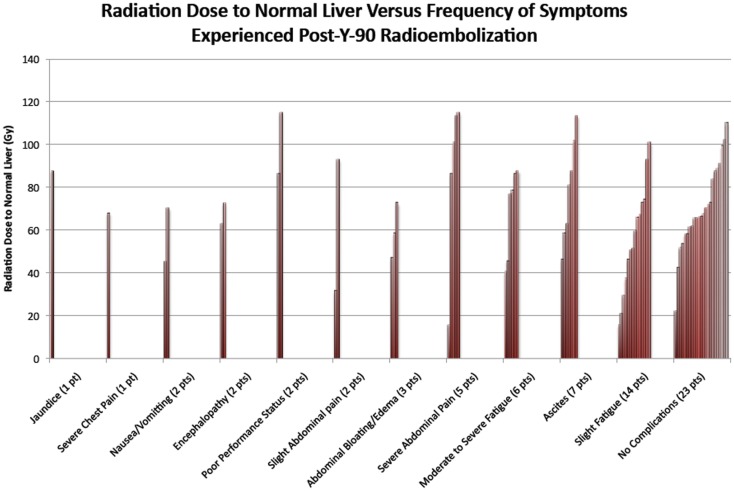
**The figure above indicates the symptoms experienced after ^90^Y radioembolization for the 56 cases evaluated in this study**. Patients in certain cases had multiple complications. Complications reported were those that had occurred within 2 months of procedure.

An additional analysis of the 56 cases in this study was done to explore a possible correlation between dose of radiation to normal liver tissue and complications suffered within 2 months of ^90^Y radioembolization (see Figure 7). The graph displays that in 23 of the 56 cases, no complications were observed in the 2 months following procedure. In the remaining 33 cases in which complications were suffered, the most common complication was slight fatigue, experienced in 14 of the cases. Ascites and moderate to severe fatigue were the next most common complications, which were experienced in seven cases and six cases, respectively. Jaundice and severe chest pain were the least common complications, which were each seen in one case. Thirty-seven had mild or no complications (i.e., slight fatigue, slight abdominal pain, and no symptoms), 15 had one severe complication, and 6 had two or more severe complications.

The absorbed dose in the normal liver varied from a low of 15 to a high of 115 Gy, with mean and median of 67. Among the 37 patients with no to mild complications, the mean absorbed dose was 65.2 Gy; among the 15 patients with one severe complication the mean absorbed dose was 64.0 Gy, and among the 6 patients with two or more severe complications, the mean absorbed dose was 87.2 Gy. There was a statistically significant association between liver absorbed dose and the presence/absence of two or more severe complications (*p* = 0.036). For patients with absorbed doses <75 Gy, the probability of two or more severe complications was 5% (2/40), the odds being 2/38; for patients with absorbed doses of 75–95 Gy, the probability was 20% (2/10), the odds being 2/8; and for patients with absorbed doses of >95 Gy, the probability was 25% (2/8), the odds being 2/6. For patients with doses of 55–75 Gy, we observed that the probability of two or more severe complications was 0.087 (2 of 23 patients), the odds were 2/21. Therefore, over the increase in Gy from 55–75 to 75–95, the risk of two or more severe complications increases 2.6 times (2/21: 2/8). From the fitted model, the odds ratio for a single unit increase in absorbed dose was 1.049, with 95% CI of [1.003, 1.096] for patients in this study with a dose range of 20–115 Gy. Statistical modeling for our cohort suggests that for every 10 Gy increase in the liver, there is an estimated 61% increase in the odds that the patient will have two or more severe complications.

## Discussion

Radioembolization is really a form of brachytherapy. Radiation oncologists, who regularly perform prostate brachytherapy with seed implants or cervical/endometrial brachytherapy with tandem and ovoids, have an understanding of the dose in Gray (Gy) delivered to tumors as well as the dose delivered to normal pelvic structures. This basic understanding is essential if one is to maximize delivery to tumor and minimize collateral damage to normal tissue. Up until now, radioembolization has been largely practiced without a strong sense for the quantity of radiation exposure to liver tumors vs. normal liver.

Lhommel, in 2009, opened a new era by demonstrating that it is possible to image the pair production positron from ^90^Y using PET/CT (26). While the biodistribution map that comes from ^90^Y PET/CT scan is itself very useful for determining the extent of treatment coverage, it is the quantitative aspect of PET that is still largely untapped. Now with the ability to quantify the amount of ^90^Y deposited in tumor and normal liver tissue regions, we can begin to understand what effect we are having on the patient. One goal is to eventually mirror that which is done in conventional brachytherapy by treating tumors to a certain level of Gy or by mandating that normal liver does not exceed a certain threshold. But even more basic than this is to understand what absorbed dose is truly considered tumoricidal and what absorbed dose can be safely administered to normal liver tissue with acceptable side effect profile. With the advent of quantitative ^90^Y PET/CT and image-based dosimetry, we can begin to address these larger questions.

Although the results of treatment responder and non-responder analysis did not prove to be statistically significant (*p* = 0.099), the data suggest a possible trend that a higher ^90^Y dose resulted in better response in those tumors able to be definitively analyzed. A larger sample size is needed to possibly reinforce this suggestion. With the above possibility in mind, the data from the analysis suggest a minimum tumor absorbed dose of around 150 Gy to get enough of a response to at least stabilize the targeted tumor. This was indicated by how stable disease response had a mean tumor absorbed dose of 154 Gy. The tumors that showed progressive disease with a mean tumor absorbed dose of 277 Gy may have been a statistical anomaly with *n* = 3, in that the high dosage may just be random occurrence. If the sample size were larger and statistical significance achieved, one could come to the conclusion that an absorbed dose >200 Gy will result in the patient being a responder. Alternatively, if no trend can be demonstrated, one may conclude that an absorbed dose >150 Gy may be sufficient for at least a tumorostatic treatment resulting in SD. Likely, all four outcomes had mean (Gy) doses in a therapeutic range, with simply aggressive tumors in the PD group. An analysis with greater numbers would help in elucidating the tumoricidal vs. tumorostatic threshold. Interestingly, our distribution of responses using mRECIST criteria is strikingly similar to that seen in the work of Strigari et al. (27).

The complications from hepatotoxicity show that dose delivered to normal liver is an important parameter to consider when treating radioembolization patients. When looking at Figure 7, one can appreciate the large range of liver dosage that patients receive. In addition, the complications vary widely, from no symptoms to severe adverse events. The idea that a threshold activity to the normal liver should not be crossed may help reduce the incidence of such events. As referenced in Strigari et al., her tolerance dose of the liver leading to 50% complication probability (TD_50_) was 52 Gy (27). In our cohort, 93% of patients received a dose of 30 Gy or larger to normal liver, with 13% of these patients experiencing some sort of complication and 11% experiencing two or more severe complications. Only one patient of four receiving a dose <30 Gy experienced any complications, though it was a severe one. It is likely that a threshold of 30 Gy to normal liver would have greatly reduced the incidence of complications.

Multiple studies have exposed the limitation of using size-based response criteria, such as RECIST, with the use of biologic targeted therapy and transcatheter therapy. In the SHARP trial, which studied the use of sorafenib in treatment of advanced HCC, up to 70% of patients who responded favorably and had a longer progression free survival had stable disease by size, but demonstrated morphologic changes such as necrosis (28). In 2000, a panel of experts on HCC convened by the European Association for the Study of the Liver (EASL) amended the response criteria to take into account tumor necrosis induced by treatment (24). Later, Lencioni and Llovet based on consensus panel of American Association for the Study of Liver Diseases and Journal of the National Cancer Institute members tweaked existing size-based criteria and found modified RECIST superior in assessing treatment response of loco-regionally treated HCC. mRECIST is based on long axis diameter measurement of the enhancing portions of the target lesions that are selected using comparable guidelines as RECIST. Likewise, response assessment categories or PD, PR, SD, and CR with percent change in the measured long axis of target lesion is also similar to that of RECIST (29). Modified RECIST has been shown to out-perform RECIST in accuracy and in its ability to predict outcomes in transcatheter treated HCC patients.

An obvious limitation to the methods presented in this paper is the fact that the activity given to normal liver is not explicitly calculated from a volume of interest. Since the normal liver volume or contour is sometimes difficult or laborious to define, we used the subdivision explained in the Section “Materials and Methods.” The effect of this methodology is that the activity to this region is averaged over the entire volume. Thus, any statistical “hot spots” or “cold spots” will not be appreciated. Of course, this is an approximation, since it is highly probable that distribution of ^90^Y microspheres to the normal liver will be heterogeneous and not homogeneous. If adverse events occur in patients who have intense focal delivery to normal liver, as opposed to some threshold value of Gy, we will not be capturing those occurrences, as our model distributes that focal activity evenly throughout the normal liver volume.

Another limitation is that HCC was the only diagnosis studied. It is possible that other types of tumors such as cholangiocarcinoma, or metastases from colorectal cancer, neuroendocrine tumors, breast cancer, uveal melanoma, prostate cancer, etc., could respond differently. Analogous types of analyses need to be performed with other tumors and metastases to see if our findings are disease specific or are generalizable across different pathologies.

## Conclusion

This study provides a simple and systematic methodology for calculation of radiation absorbed dose for both tumors and normal liver in ^90^Y radioembolization. The authors believe that quantitative ^90^Y PET/CT image-based dosimetry provides a greater understanding of tumor response and the subsequent clinical outcomes. Our cohort of patients showed a possible dose–response trend for the tumors, and complications resulting from dose to normal liver. These methods in the future can provide the tools to help understand whether patient adverse events, treatment success, or treatment failure can be attributed to the dose that the tumor or normal liver received.

## Conflict of Interest Statement

The authors declare that the research was conducted in the absence of any commercial or financial relationships that could be construed as a potential conflict of interest.
